# Comparison of atopy patch testing to skin prick testing for diagnosing mite-induced atopic dermatitis: a systematic review and meta-analysis

**DOI:** 10.1186/s13601-017-0178-3

**Published:** 2017-11-29

**Authors:** Yumei Liu, Jianglong Peng, Ying Zhou, Yubao Cui

**Affiliations:** 1 School of Public Health, Hainan Medical University, Haikou, 571101 People’s Republic of China; 2 School of Tropical and Laboratory Medicine, Hainan Medical University, Haikou, 571101 People’s Republic of China; 3 Department of Pediatrics Laboratory, Wuxi People’s Hospital Affiliated to Nanjing Medical University, Wuxi, 214023 People’s Republic of China; 4 Department of Clinical Laboratory, Wuxi People’s Hospital Affiliated to Nanjing Medical University, No. 299 at Qingyang Road, Wuxi, 214023 Jiangsu Province People’s Republic of China

**Keywords:** Atopic eczema/dermatitis syndrome, Atopy patch test, Skin prick test, Diagnosis

## Abstract

**Background:**

Atopic dermatitis (AD) can occur after contact with aeroallergens like house dust mites, pollen, and animal dander. Despite its controversial diagnostic value, the atopy patch test (APT) has been used as an important tool in the diagnosis of AD caused by house dust mites. Here, we present a meta-analysis comparing APT to the common skin prick test (SPT) in the diagnosis of mite-induced AD.

**Methods:**

A structured search was performed using online databases and bibliographies published as of April 30, 2017. All studies evaluating the accuracy of APT and SPT in the diagnosis of mite-induced atopic eczema/dermatitis syndrome were selected, appraised, and data was extracted.

**Results:**

Ten studies were identified for inclusion in our analysis. Meta-analysis revealed that the pooled sensitivity, specificity, positive likelihood ratio, negative likelihood ratio, and diagnostic odds ratios for APT were 0.54 (95% CI 0.42–0.66), 0.72 (95% CI 0.56–0.85), 1.97 (95% CI 1.20–3.23), 0.63 (95% CI 0.48–0.83), and 3.12 (95% CI 1.53–6.39). The area under the summary receiver operating characteristic curve was 0.65 (95% CI 0.61–0.69).

**Conclusions:**

Our analysis indicates that APT is a useful tool in the screening of mite-induced AD, although this conclusion must be interpreted cautiously due to high heterogeneity among the included studies.

## Background

Atopic dermatitis (AD) is a common, chronic, relapsing inflammatory skin disease with frequencies ranging from 10 to 45% depending on the study population [1, 2]. AD, also known as atopic eczema and intrinsic allergic dermatitis, was first defined in 1930 as a condition similar to other atopic diseases like bronchial asthma (AB) and allergic rhinitis (AR) [3]. In 1980, Hanifin and Rajka formally described the AD diagnostic criteria and in 1993, the SCORAD system (SCORing atopic dermatitis) was introduced to determine the clinical severity of AD based on the intensity and extent of eczematous skin reactions [4–6]. Both diagnostic methods are universally accepted and widely used in clinical practice. In 2001, a new classification based on the pathomechanisms of skin lesion development was introduced by the European Academy of Allergy and Clinical Immunology (EAACI), which considers AD a syndrome composed of both allergic AD (associated or not associated with elevated IgE) and non-allergic AD [7].

Airborne allergens, such as domestic mites (house dust and storage mites), plant pollen allergens, animal epithelia, and molds are known to aggravate skin lesions in AD patients [8]. Accordingly, most patients with AD have high concentrations of total and allergen-specific serum IgE levels and react positively to immediate skin prick and intra-cutaneous tests involving common environmental allergens. Skin tests and allergen-specific serum IgE are widely used to assess type I hypersensitivity in both respiratory and skin allergies. As proposed by the EAACI [9], AD patients may display a delayed (type IV) or a mixed immediate and delayed (type I and IV) response, with the eczematous reaction at the site of application after 48–72 h determining the sensitization of the patient to the allergen [10]. Together with a history of atopy, clinical physical examination of AD flare-ups following skin tests, and/or allergen-specific serum IgE levels, type I but not type IV hypersensitivity can be determined in the etiopathogenesis of AD and cannot account for reactivity related to aeroallergen intolerance [11]. Atopy patch testing (APT) was first described by Mitchell [12], involving the application of the suspected allergen directly to the skin using the same method of patch testing used for contact dermatitis. APT has been shown to be an important tool in screening allergens in AD patients [13], since positive reactions are rarely observed without a skin prick test or allergen-specific IgE antibodies to aeroallergens [14]. Here, we provide a systematic review and meta-analysis comparing APT to skin prick tests (SPT) in the diagnosis of AD.

## Methods

### Literature search and study identification

We performed a literature search of PubMed, the Cochrane Library, EMBASE, Medion, and Web of Science databases to identify eligible studies published before April 30, 2017. Various combinations of medical subject headings (MeSH) and non-MeSH terms were used as follows: (dust mite or storage mite) or (domestic mite) and (sensitization or allergy or hypersensitivity or specific IgE positive or skin test positive or RAST positive) and (patch test or patch testing). In addition to published studies in these electronic databases, a manual search of related reports from major annual meetings in the field of pediatrics and reference sections of studies as well as relevant reviews was also performed. Inclusion criteria for eligible studies were as follows: (1) diagnostic accuracy and test design; (2) the index test used in the study was a patch test; (3) the reference test was a skin prick test; (4) the minimum number of study subjects was ten; and (5) a two-by-two contingency table could be constructed for mite allergy diagnosis with the index and reference test from the data presented in the study. Studies were excluded if they met the following criteria: (1) studies were conducted on animals or in vitro systems; (2) the article was a review, case report, or editorial comment; (3) patch testing was used in the diagnosis of atopic dermatitis without using mite extract; (4) 2 × 2 table construction was impossible for mite allergy diagnosis; (5) the reference test was specific IgE detection with mite extract; (6) Not for atopic dermatitis only; or (7) studies contained overlapping participants. Notably, articles by the same author or research group were included only when a different sample of patients was used. Two investigators independently performed the literature search and study identification according to the Preferred Reporting Items for Systematic reviews and Meta-Analyses (PRISMA) statement [15]. Any disagreement was resolved by discussion between the two reviewers.

### Quality assessment

To assess the quality of each included study, we used the Quality Assessment of Diagnostic Accuracy Studies (QUADAS-2) tool [16]. Briefly, QUADAS-2 comprises four key domains: patient selection, an index test, reference test flow of patients through the study, and the timing of the index and reference tests (flow and timing). These four domains were used to evaluate the risk of bias and the first three were applied to assess applicability. According to the investigators’ answers for all signaling questions in each domain, risks of bias were graded as “low risk”, “high risk”, or “unclear risk”. To address applicability concerns, review authors documented relevant information and assessed whether the study matched the review question. Concerns of applicability were rated as “low risk”, “high risk”, or as having “unclear risk”. A standardized table and figure, recommended by the Quality Assessment of Diagnostic Accuracy Studies (QUADADS-2) website, were used to display the summarized results of the QUADAS-2 with the number of studies observed with low, high, or unclear risk of bias or applicability concerns for each domain.

### Data extraction

Characteristic information from the selected studies was extracted, including year of publication, country of origin, study design, patient characteristics, number of study participants, diagnostic criteria, and reference tests for the diagnosis of mite-induced atopic dermatitis. Absolute numbers of true positives (TP), false positives (FP), false negatives (FN), and true negatives (TN) was also extracted.

### Diagnostic measures combination

The pooled sensitivity, specificity, positive likelihood ratio (PLR), negative likelihood ratio (NLR), diagnostic odds ratio (DOR), diagnostic score, and area under the summary receiver-operating curve (AUSROC) with the corresponding 95% confidence interval (CI) were obtained using a bivariate binomial mixed model [17]. The sensitivity, specificity, DOR, and AUSROC were considered to be the major outcomes of this analysis.

### Heterogeneity

A Cochrane-Q test of heterogeneity was performed using the inconsistency index, *I* ^2^, as a measure to illustrate the percentage of the total variability among trials caused by heterogeneity instead of chance [18]. A value of *I* ^2^ more than 50% designated heterogeneity. A two-sided p value < 0.05 indicated statistical significance.

### Diagnostic threshold effects

Since the cut-off values were different among the included studies, diagnostic threshold effects were inspected [19]. First, the summary receiver-operating curve (SROC) was visually evaluated. Then, a Spearman correlation analysis was used to assess the heterogeneity derived from diagnostic threshold effects.

### Publication bias

Deeks’ funnel plot asymmetry analysis was performed to identify publication bias [19]. Briefly, a Deeks’ funnel plot was created as a scatter plot of the inverse of the square root of effective sample size [1/root (ESS)] against the ln (DOR).

### Fagan’s nomogram analysis

A Fagan’s nomogram plot was constructed, comprising three vertical axes [20]. The left axis represented pre-test probability derived from the prevalence in each included study. Another axis in the middle displayed the likelihood ratio showing the extent to which the index could raise or lower the probability of having the disease. The right vertical axis signified the post-test probability of a patient having the positive or negative results of the reference standard test after the index test result was known.

### Bivariate boxplot

With logit specificity and logit sensitivity as the horizontal and vertical axes, respectively, a bivariate boxplot was applied to assess the distributional properties of sensitivity against specificity and investigate possible outliers [21].

### Data synthesis and statistical analysis

Data synthesis and most statistical analyses were undertaken using STATA software version 12.0 (College Station, TX, USA).

## Results

### Literature search results and trial characteristics

**Fig. 1:**
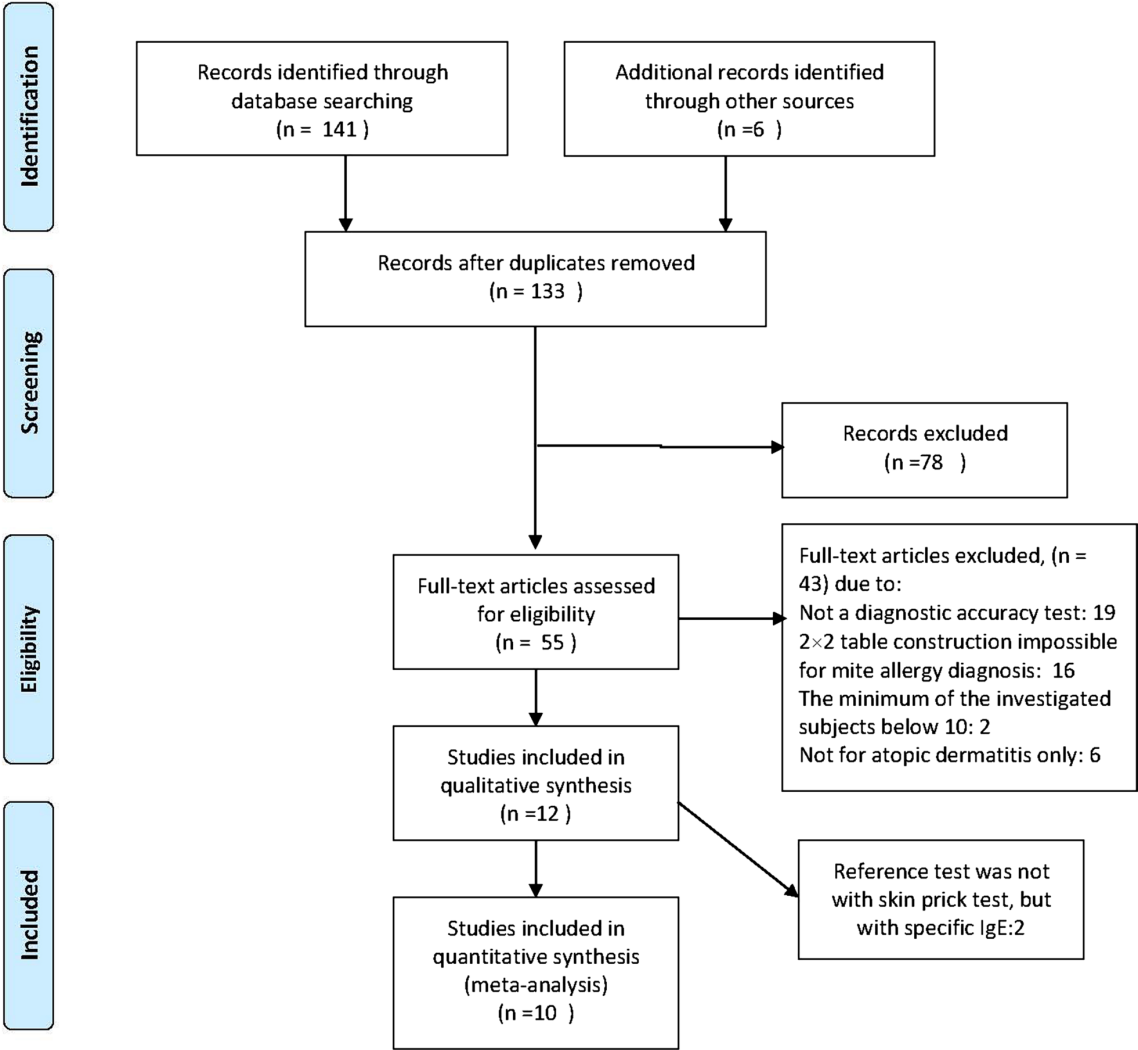
Flow chart depicting the search and selection strategy for eligible studies. *n* number of studies

The initial search identified 141 references from PubMed, the Cochrane Library, EMBASE, Medion and Web of Science databases, along with six additional reports resulting from screening review article references. Since the search strategy was relatively broad, most of the results were not eligible. After screening titles and abstracts, 14 studies were identified as duplicates and 78 studies were excluded. After full-text assessment, ten studies were included for original data, clarification of methods, and meta-analysis [22–31]. Figure 1 illustrates our method of study inclusion. Characteristics of included studies and patient baseline demographics are displayed in Table 1.

**Table 1:** Characteristics of eligible studies

Study	Country	Cohort age (mean ± SD years) (range)	Number of patients (n, female/male)	Mite species (supplier)	TP	FP	FN	TN
Heinemann et al. [23]	Germany	25.4 (18–43)	52 (37/15)	*D. pteronyssinus* (ALK-Scherax-Arzneimittel)	13	1	10	28
Wistokat-Wülfing et al. [24]	Germany	NR	32 (NR)	*D. pteronyssinus* (ALK-Scherax-Arzneimittel)	14	3	7	8
Lorenzini et al. [25]	Brazil	9.47 (2–31)	43 (NR)	*A. ovatus* (in house)	0	6	7	30
Darsow et al. [26]	Germany	29 ± 16 (3-69)	36 (27/9)	*D. pteronyssinus* (Bencard, Allergopharma)	8	5	12	11
Kutlu et al. [31]	Turkey	6.9 ± 3.6 (2– 15)	44 (23/21)	*D. pteronyssinus* (ALK-Abello, Stallergėnes)	7	2	11	24
Manzini et al. [27]	Italy	(0.5– 63)	313 (NR)	*D. pteronyssinus* and *D. farina* (Bayropharm, Lofarma, Allergopharma	46	76	54	137
Wananukul et al. [28]	Thailand	(2–14)	30 (NR)	*D. pteronyssinus* (GREER)	21	0	8	1
Michel et al. [29]	Switzerland	35.8 (14–73)	23 (16/7)	*D. pteronyssinus* (Stallergėnes*)	6	5	5	7
Holm [22]	Sweden	29 (18–65)	81 (54/27)	*D. pteronyssinus and D. farina* (ALK-Abello)	14	23	5	39
Fuiano et al. [30]	Italy	2.2 ± 2.5	15 (NR)	*Dermatophagoides* (Stallergėnes*)	1	11	0	3

*NR* not reported. * Stallergėnes merged with GREER in 2015

Among the ten trials published between 1993 and 2015, two studies were conducted in Asian countries (Thailand [28] and Turkey [31]), one study was conducted in South America (Brazil [25]), while the remaining seven were conducted in European countries (Germany [23, 24, 26], Italy [27, 30], Switzerland [29], and Sweden [22]). The sample size of each study ranged from 15 to 313 patients, with only one study involving 313, and the other nine having no more than 100 patients. Four studies focused on children [25, 28, 30, 31]; the others included patients of all ages. For diagnosis, the majority of studies used the criteria of Hanifin and Rajka [4]. Two studies [27, 28] used that criteria alone, three [24, 25, 30] combined it with SCORAD, and one [29] combined it with SCORAD and the atopy score of Diepgen et al. [32]. SCORAD [5, 6], which summarizes items by the intensity and extent of the eczematous skin reaction, was used alone in two studies [26, 31] and with clinical history in one [22]. One study [23] relied on clinical history and the Erlangen atopy score, which is the same as the Diepgen score [32], for diagnosis. All of the trials used a skin test as the reference standard. The count data for primary studies including true positive (TP), false positive (FP), false negative (FN), and true negative (TN) were extracted and are presented in Table 1.

**Fig. 2:**
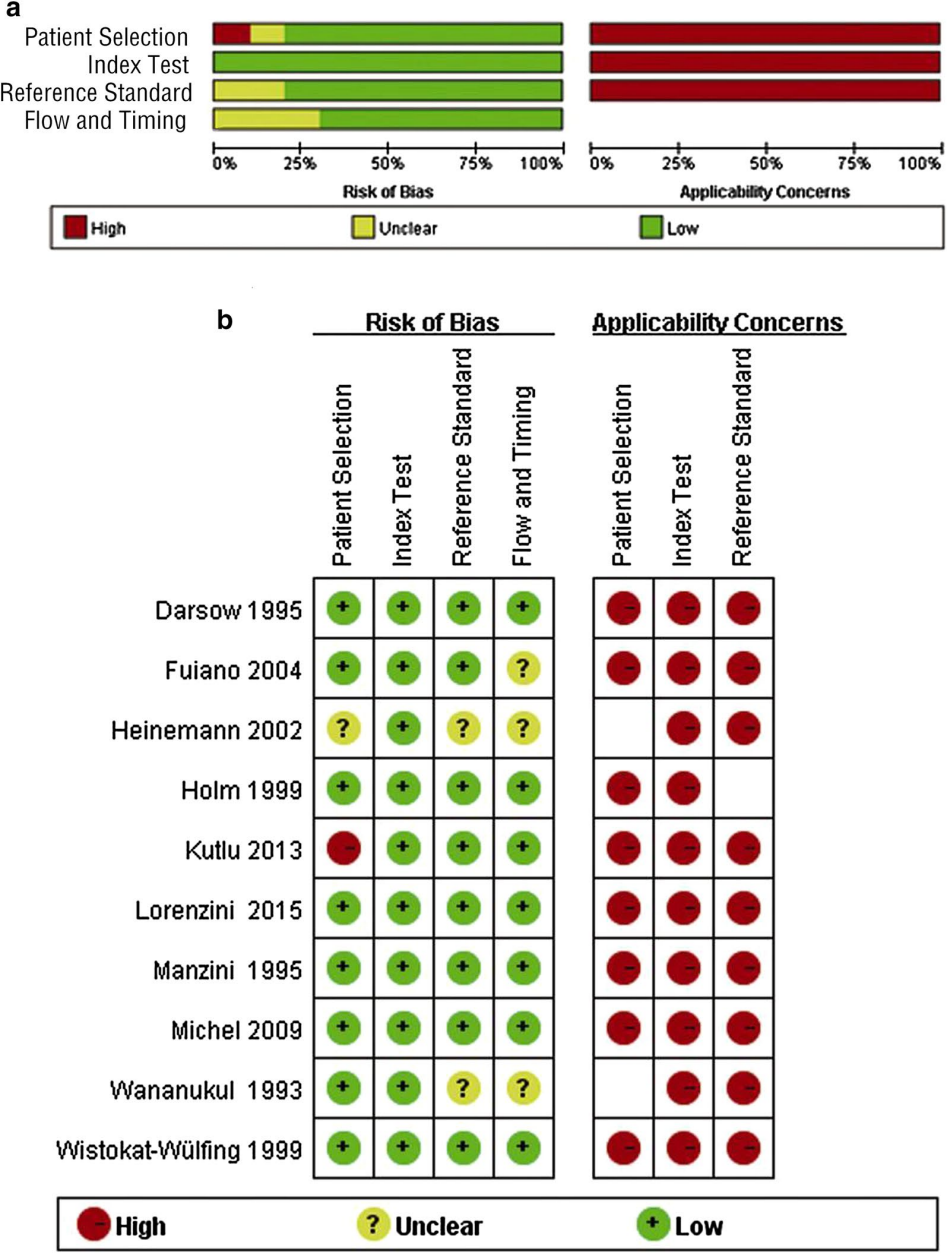
Cumulative bar plot (**a**) and summary table (**b**) of risk of bias and applicability concerns across all studies

**Fig. 3:**
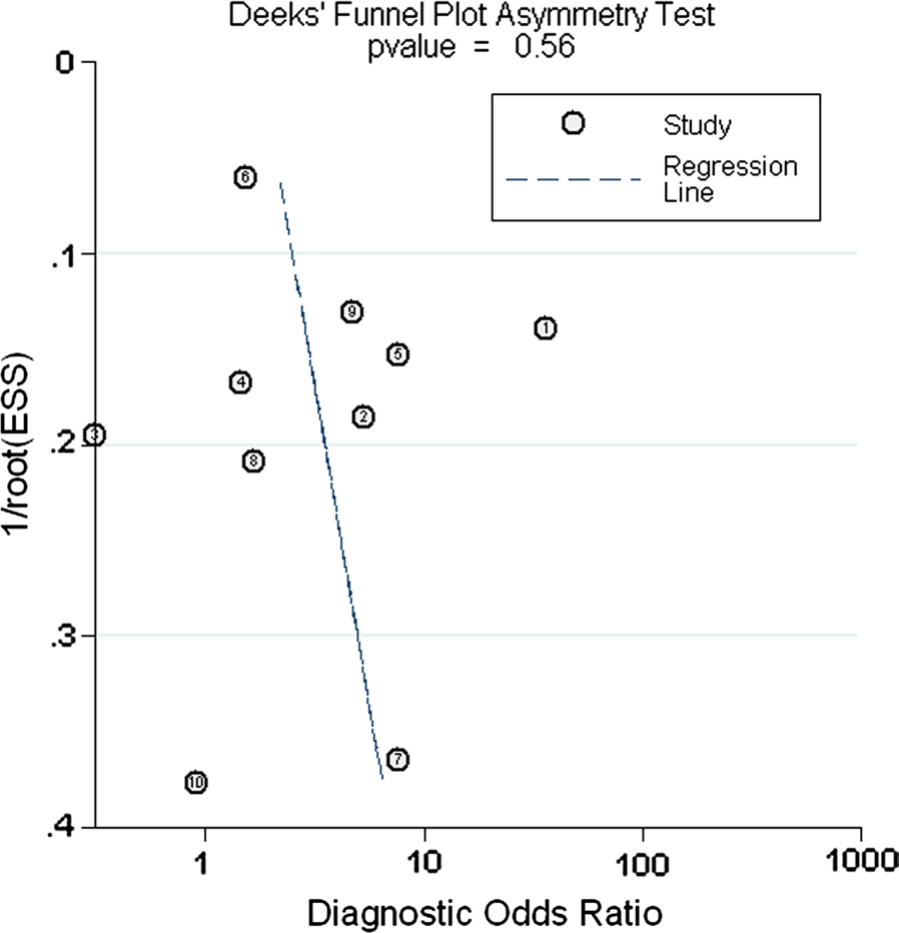
Deeks’ funnel plot for detecting publication bias. ① Heinemann et al.; ② Wistokat-Wülfing et al.; ③ Lorenzini et al.; ④ Darsow et al.; ⑤ Kutlu et al.; ⑥ Manzini et al.; ⑦ Wananukul et al.; ⑧ Michel et al.; ⑨ Holm et al.; ⑩ Fuiano et al

**Fig. 4:**
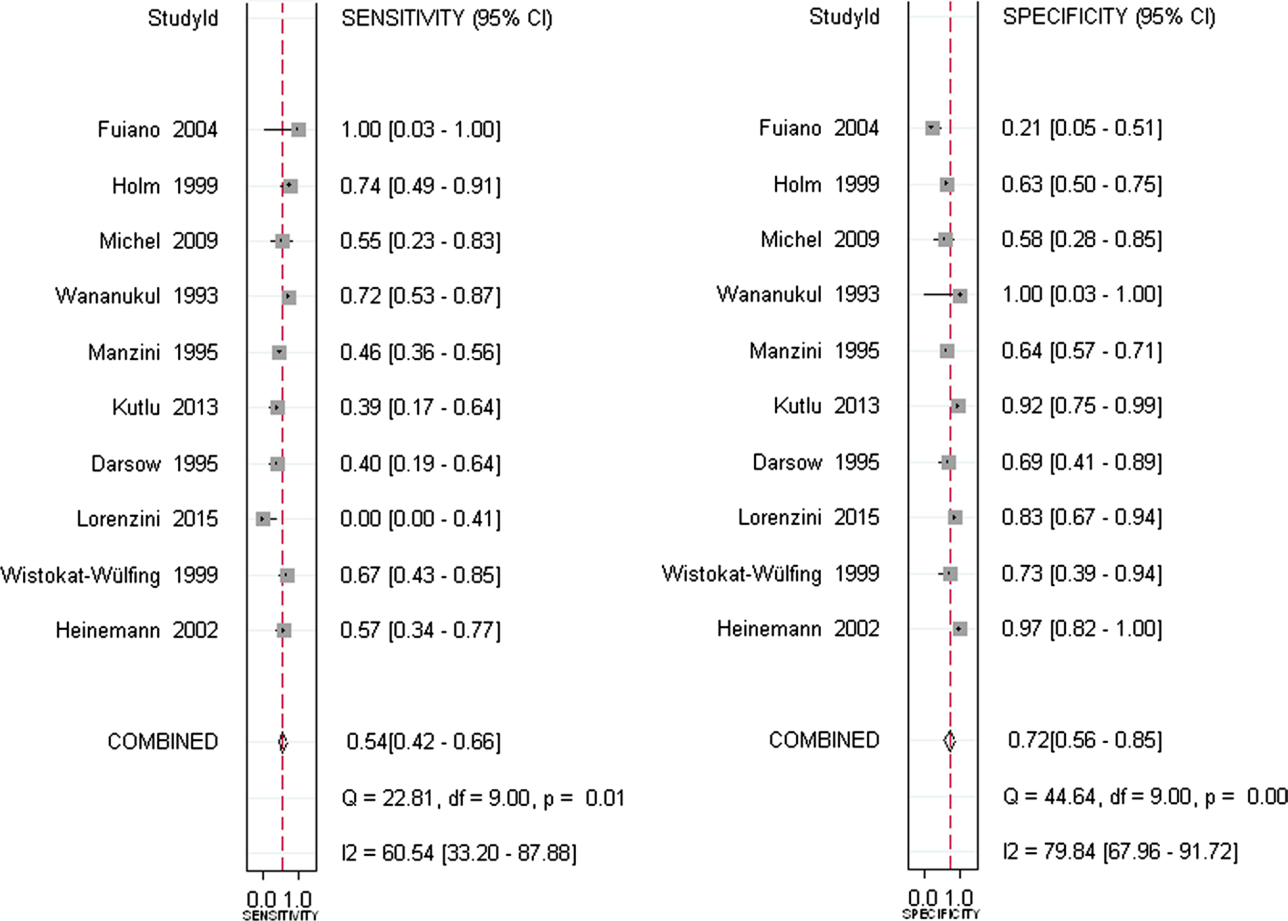
Forest plot of sensitivity and specificity of APT in comparison to SPT for the diagnosis of patients with atopy dermatitis. *CI* confidence interval, *ES* estimates

Using the criteria of Quality Assessment of Diagnostic Accuracy Studies (QUADAS-2), an updated evaluation tool for the systematic review and meta-analysis of diagnostic test accuracy, the cumulative bar plot and summary of bias risk and applicability concerns are shown in Fig. 2, summarizing the quality conditions across studies. Quality evaluation was performed independently by two investigators. As shown, the quality of the ten eligible studies was not significantly affected by bias. A lack of bias is also evident in a Deeks’ funnel plot (Fig. 3). The plot has a symmetrical funnel shape, indicating that publication bias was likely absent. Furthermore, the *p* value for the Deeks’ funnel plot asymmetry test was 0.56, indicating a lack of publication bias in this meta-analysis. However, there was substantial heterogeneity among the ten studies (Q = 14.744, p = 0.000; overall *I* ^2^ for bivariate model 86, 95% CI 72–100), as demonstrated in Fig. 4.

### Data synthesis of diagnostic accuracy

**Fig. 5:**
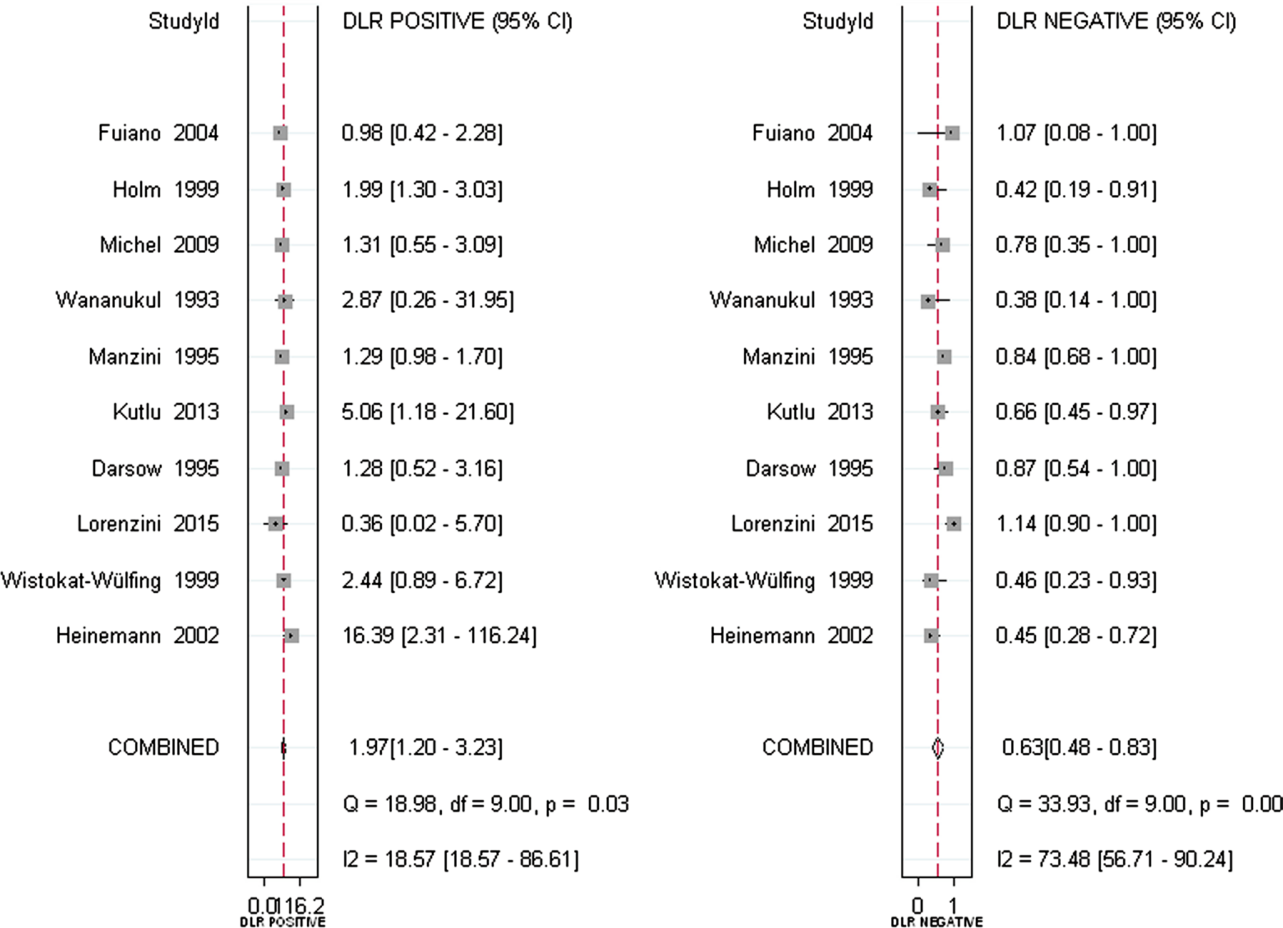
The pooled PLR and NLR of APT in comparison to SPT for the diagnosis of patients with atopy dermatitis. *CI* confidence interval, *ES* estimates

**Fig. 6:**
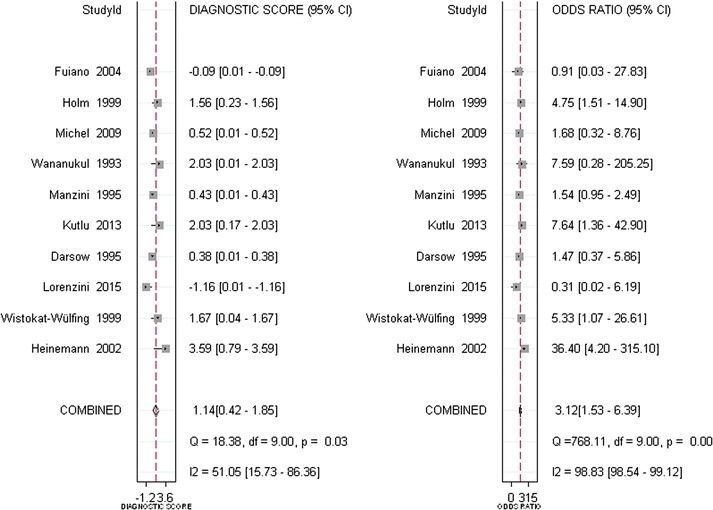
The combined diagnostic score and odds ratio (OR) of APT in comparison to SPT for the diagnosis of patients with atopy dermatitis. *CI* confidence interval, *ES* estimates

**Fig. 7:**
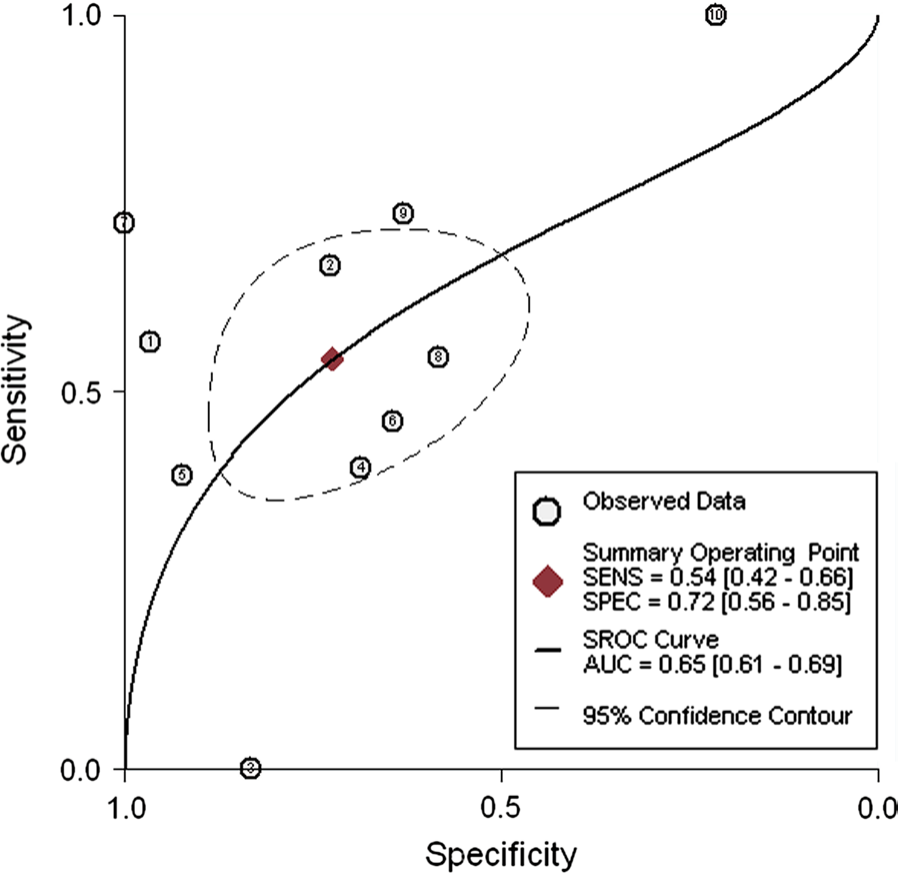
Summary receiver operating curve of APT in comparison to SPT for the diagnosis of patients with atopy dermatitis. AUC, area under curve; SROC, summary receiver operating curve; SENS, sensitivity; SPEC, specificity. ① Heinemann et al.; ② Wistokat-Wülfing et al.; ③ Lorenzini et al.; ④ Darsow et al.; ⑤ Kutlu et al.; ⑥ Manzini et al.; ⑦ Wananukul et al.; ⑧ Michel et al.; ⑨ Holm et al.; ⑩ Fuiano et al

**Fig. 8:**
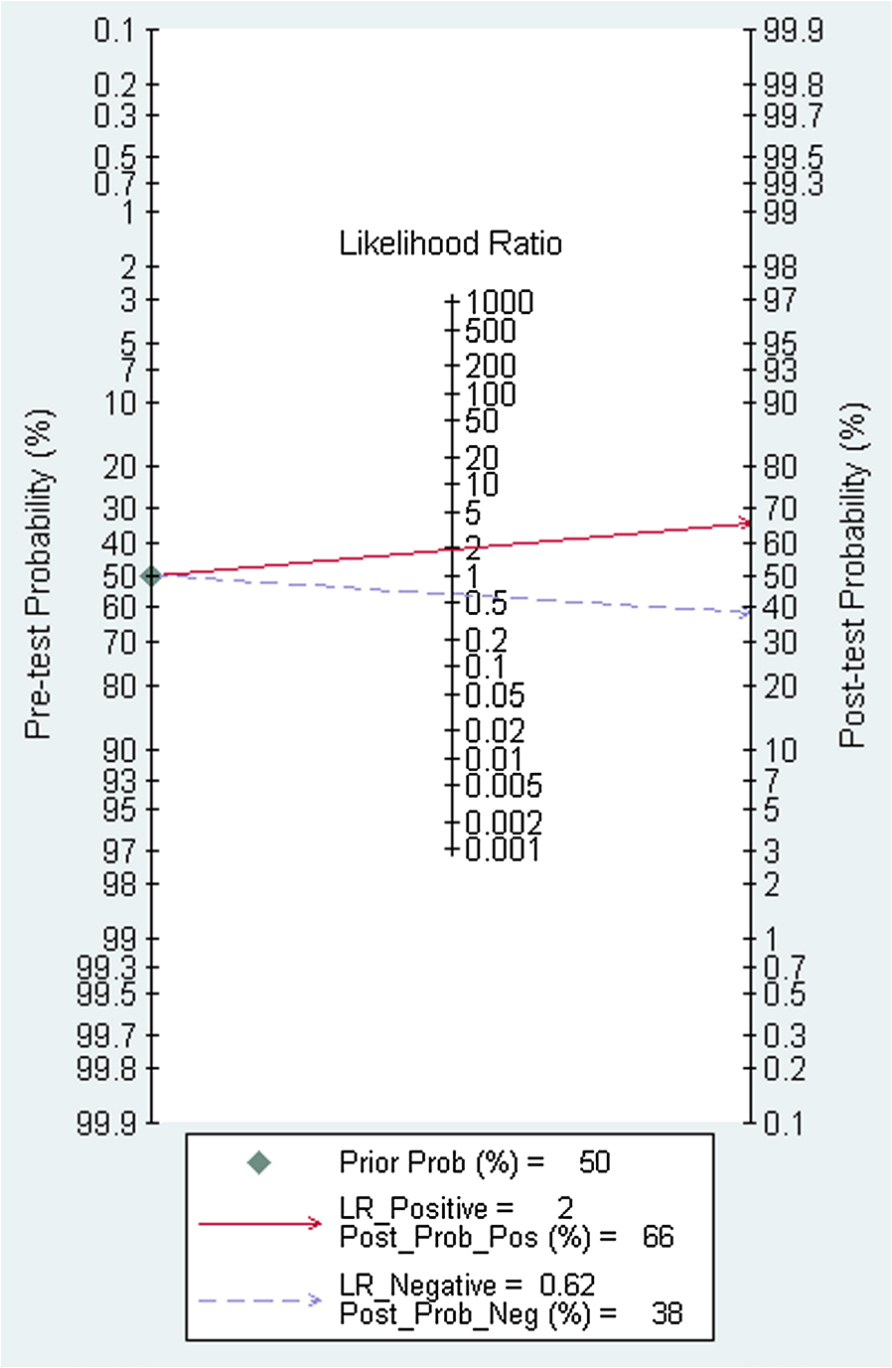
Fagan’s nomogram plot to evaluate the clinical utility of APT in comparison to SPT for the diagnosis of patients with atopy dermatitis. The vertical axis on the left displays a fixed pre-test probability of 50%. Using the likelihood ratio in the middle axis, post-test probability (patient’s probability of having the disease after the index test result was known) was obtained

In total, 669 subjects from ten studies were included in our systematic review and meta-analysis. With a bivariate model, diagnostic performances of APT in Atopic eczema/dermatitis syndrome were pooled and are summarized in Table 2. The combined estimates of sensitivity and specificity for APT compared to SPT in the diagnosis of AEDS were 0.54 (95% CI 0.42–0.66) and 0.72 (95% CI 0.56–0.85), respectively (Table 2 and Fig. 4). The corresponding summary PLR and NLR were 1.97 (95% CI 1.20–3.23) and 0.63 (95% CI 0.48–0.83), respectively (Table 2 and Fig. 5). The combined diagnostic score and odds ratio (OR) were 1.14 (95% CI 0.42–1.85) and 3.12 (95% CI 1.53–6.39) (Table 2 and Fig. 6). Figure 7 shows that the area under the SROC was 0.65 (95% CI 0.61–0.69). Fagan’s Nomogram analysis (Fig. 8) revealed that, with a fixed pre-test probability of 50% and a pooled PLR of 1.97, the post-test probability was increased to 66%. Conversely, with a combined NLR of 0.63, the post-test probability was decreased to 39%.

**Table 2:** Summary of the pooled estimates of studies using APT in comparison to SPT for the diagnosis of patients with atopy dermatitis

	ES [95% CI]
Number of included studies	10
Number of subjects	669
Sensitivity	0.54 [0.42, 0.66]
Specificity	0.72 [0.56, 0.85]
Positive likelihood ratio	1.97 [1.20, 3.23]
Negative likelihood ratio	0.63 [0.48, 0.83]
Diagnostic odds ratio	3.12 [1.53, 6.39]
AUSROC	0.65 [0.61–0.69]

AUSROC, area under summary receiver operating curve; *ES* estimates, *CI* 95% confidence intervals

**Fig. 9:**
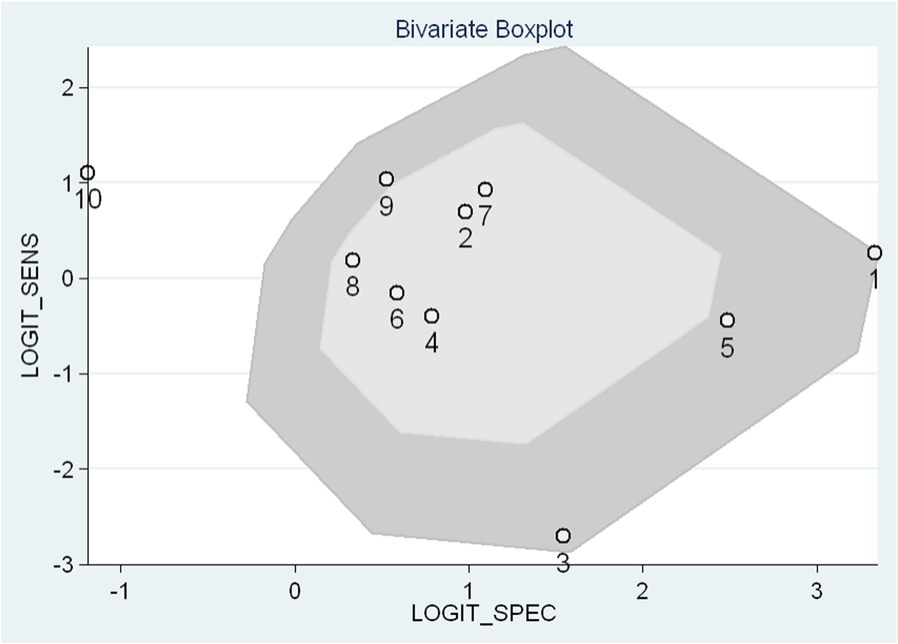
Bivariate box plot for evaluating outliers. ① Heinemann et al.; ② Wistokat-Wülfing et al.; ③ Lorenzini et al.; ④ Darsow et al.; ⑤ Kutlu et al.; ⑥ Manzini et al.; ⑦ Wananukul et al.; ⑧ Michel et al.; ⑨ Holm et al.; ⑩ Fuiano et al

To evaluate the distributional properties of sensitivity versus specificity and identify possible outliers, a bivariate box plot analysis was used. As shown in Fig. 9, the data from the study by Fuiano et al. [30] and Heinemann et al. [23] reached or nearly reached the limit of extreme value, indicating that both studies are potentially heterogeneous with regard to the other studies. In addition, data from three studies, Lorenzini et al. [25], Kutlu et al. [31], and Holm et al. [22], were mild outliers. The shape of the bivariate box plot was symmetrical, indicating that the data has a normal distribution.

## Discussion

Aeroallergen contact is an important etiologic factor in skin allergy as skin symptoms typically worsen with allergen contact and improve with allergen avoidance [33]. This sometimes necessitates the removal of patients from their typical environments. Importantly, allergen-specific immunotherapy with house dust mites can significantly improve symptoms in patients with severe AD [33]. SPT is simple, inexpensive, and the results are immediately available hence is usually the preferred method for identifying allergens in patients with IgE-mediated hypersensitivity. However, diagnostic approaches are rather complex for late onset reactions, because the role of allergens in the pathogenesis and clinical features of AD have not been explored in detail. For example, increasing evidence indicates that T-cell responses to environmental allergens have an important role in the pathogenesis of atopic dermatitis [24].

APT with allergens can induce delayed sensitization at the testing site and was introduced to assess sensitization to inhalant allergens in patients with AD. This systematic review and meta-analysis of ten studies, including 669 cases, provides an overview of the diagnostic performances (pooled sensitivity, specificity, PLR, NLR, and DOR) of APT relative to SPT for diagnosing AD (Table 2). It is well known that the area under the SROC provides a holistic estimation of diagnostic accuracy. According to the recommended guidelines for the interpretation of AUSROC values [34], the diagnostic ability of APT in determining AD was moderate [AUSROC: 0.65 (95% CI 0.61–0.69)].

For this meta-analysis, SPT was considered the reference test. Positive APT responses are usually found in people with highly specific IgEs, but these metrics can be dissociated [35]. In our initial literature search, two studies were excluded due to reference tests with specific IgEs [36]. One was reported by Imayama and coworker [37] and showed no correlation between serum IgE levels and the ATP reactions for dust mite allergens; the other was reported by Langeveld-Wildschut et al. [38] and reported significantly higher allergen-specific IgE levels in a group of patients with AD and positive APT results.

In the ten studies we analyzed, the percentage of ATP-positive subjects ranged from 14-70%, likely due to the lack of standardized techniques and dissimilarities in allergen source and purification in both the APT and SPT tests. Commercial material was typically used for both APT and SPT, but the supplier varied (see Table 1) and one study derived their own extracts [25]. The majority of studies used *D. pteronyssinus*, but a few studies examined other mite species (see Table 1). Whole mite bodies, extracts, and/or purified major mite allergens were applied in different vehicles (PBS or petrolatum) using different sizes of Finn chambers. The length of exposure and the concentrations of allergen extracts also varied depending on the study, and various methods were used to score APT results, including criteria established by Wahlberg [39], Darsow et al. [26], or the International Contact Dermatitis Research Group [40]. Studies also used scoring protocols described in Turjanmaa et al. [41], Nicol et al. [42] or Sertoli et al. [43]. Therefore, the substantial heterogeneity observed in this analysis (Q = 14.744, p = 0.000; overall *I* ^2^ for bivariate model 86, 95% CI 72–100) was likely associated with the different sources of allergen extract employed by the studies and the lack of standardized APT techniques. However, publication bias was not identified in this meta-analysis.

## Conclusions

We conclude that atopy patch testing is suitable for identifying mite-sensitization in patients with atopy dermatitis and should be used alongside SPT. However, since the positive response rate varied based on the type of allergen material, the choice of allergenic extract remains an impactful and critical factor in determining AD. Aside from better standardization and an improved definition of mite-derived material, a multicenter comparison of different extracts and their diagnostic value in atopic and healthy subjects would be valuable.

## References

[1] Dou X, Kim J, Ni CY, Shao Y, Zhang J (2016). Atopy patch test with house dust mite in Chinese patients with atopic dermatitis. J Eur Acad Dermatol Venereol.

[2] Al-Saimary IE, Al-Hamdi KE, Bakr SS (2013). The prevalence of atopic eczema/dermatitis syndrome (AEDS) in Basrah Providence, IRAQ. Advances in Bioresearch..

[3] Wüthrich B, Schmid-Grendelmeier P (2003). The atopic eczema/dermatitis syndrome. Epidemiology, natural course, and immunology of the IgE-associated (“extrinsic”) and the nonallergic (“intrinsic”) AEDS. J Investig Allergol Clin Immunol.

[4] Hanifin J, Rajka G (1980). Diagnostic features of atopic eczema. Acta Derm Venereol.

[5] Listed N (1993). Severity scoring of atopic dermatitis: the SCORAD index. Consensus report of the European Task Force on Atopic Dermatitis. Dermatology.

[6] Stalder JF, Taïeb A (1993). Severity scoring of atopic eczema: the SCORAD Index. Dermatology.

[7] Bruynzeel-Koomen CA, Van Wichen DF, Spry CJ, Venge P, Bruynzeel PL (1988). Active participation of eosinophils in patch test reactions to inhalant allergens in patients with atopic dermatitis. Br J Dermatol.

[8] Czarnecka-Operacz M, Bator-Wegner M, Silny W (2005). Atopy patch test reaction to airborne allergens in the diagnosis of atopic dermatitis. Acta Dermatovenerol Croat ADC.

[9] Samochocki Z, Owczarek W, Zabielski S (2006). Can atopy patch tests with aeroallergens be an additional diagnostic criterion for atopic dermatitis?. Eur J Dermatol.

[10] Fabrizi G, Romano A, Vultaggio P, Bellegrandi S, Paganelli R, Venuti A (1999). Heterogeneity of atopic dermatitis defined by the immune response to inhalant and food allergens. Eur J Dermatol Ejd..

[11] Novak N, Bieber T (2003). Allergic and nonallergic forms of atopic diseases. J Allergy Clin Immu.nol.

[12] Mitchell EB, Crow J, Chapman MD, Jouhal SS, Pope FM, Platts-Mills TA (1982). Basophils in allergen-induced patch test sites in atopic dermatitis. Lancet.

[13] Fuiano N, Incorvaia C (2011). The atopy patch test: Is it time to redefine its significance?. Ann Allergy Asthma Immunol.

[14] Ring J, Darsow U, Gfesser M, Vieluf D (1997). The ‘atopy patch test’ in evaluating the role of aeroallergens in atopic eczema. Int Arc Allergy Immunol.

[15] Liberati A, Altman DG, Tetzlaff J, Mulrow C, Gotzsche PC, Ioannidis JP (2009). The PRISMA statement for reporting systematic reviews and meta-analyses of studies that evaluate healthcare interventions: explanation and elaboration. Epidemiol Biostat Public Health.

[16] Whiting PF, Rutjes AW, Westwood ME, Mallett S, Deeks JJ, Reitsma JB (2011). QUADAS-2: a revised tool for the quality assessment of diagnostic accuracy studies. Ann Intern Med.

[17] Menke J (2013). Bivariate random-effects meta-analysis of sensitivity and specificity with the Bayesian SAS PROC MCMC: methodology and empirical evaluation in 50 meta-analyses. Med Decis Making.

[18] Devillé WL, Buntinx F, Bouter LM, Montori VM, de Vet HC, van der Windt DA (2002). Conducting systematic reviews of diagnostic studies: didactic guidelines. BMC Med Res Methodol.

[19] Deeks JJ, Macaskill P, Irwig L (2005). The performance of tests of publication bias and other sample size effects in systematic reviews of diagnostic test accuracy was assessed. J Clin Epidemiol.

[20] Caraguel CGB, Vanderstichel R (2013). The two-step Fagan’s nomogram: ad hoc interpretation of a diagnostic test result without calculation. Evid Based Med.

[21] Reitsma JB, Glas AS, Rutjes AW, Scholten RJ, Bossuyt PM, Zwinderman AH (2005). Bivariate analysis of sensitivity and specificity produces informative summary measures in diagnostic reviews. J Clin Epidemiol.

[22] Holm L, van Hage-Hamsten M, Öhman S, Scheynius A (2015). Sensitization to allergens of house-dust mite in adults with atopic dermatitis in a cold temperate region. Allergy.

[23] Heinemann C, Schliemann-Willers S, Kelterer D, Metzner U, Luge K, Wigger-Alberti W (2002). The atopy patch test—reproducibility and comparison of different evaluation methods. Allergy.

[24] Wistokat-Wülfing A, Schmidt P, Darsow U, Ring J, Kapp A, Werfel T (1999). Atopy patch test reactions are associated with T lymphocyte-mediated allergen-specific immune responses in atopic dermatitis. Clin Exper Allergy.

[25] Lorenzini D, Pires M, Aoki V, Takaoka R, Souza RL, Vasconcellos C (2015). Atopy patch test with *Aleuroglyphus ovatus* antigen in patients with atopic dermatitis. J Eur Acad Dermatol Venereol.

[26] Darsow U, Vieluf D, Ring J (1995). Atopy patch test with different vehicles and allergen concentrations. J Allergy Clin Immunol.

[27] Manzini BM, Motolese A, Donini M, Seidenari S (1995). Contact allergy to Dermatophagoides in atopic dermatitis patients and healthy subjects. Contact Dermat.

[28] Wananukul S, Huiprasert P, Pongprasit P (1993). Eczematous skin reaction from patch testing with aeroallergens in atopic children with and without atopic dermatitis. Pediatr Dermatol.

[29] Michel S, Yawalkar N, Schnyder B, Fischer B, Helbling A (2009). **Eczematous sakin reaction to atopy patch testing with cockroach in patients with atopic dermatitis. J Invest Allergol Clin Immunol.

[30] Fuiano N, Incorvaia C (2004). Value of skin prick test and atopy patch test in mite-induced respiratory allergy and/or atopic eczema/dermatitis syndrome. Minerva Pediatr.

[31] Kutlu A, Karabacak E, Aydin E, Ozturk S, Taskapan O, Aydinoz S (2013). Relationship between skin prick and atopic patch test reactivity to aeroallergens and disease severity in children with atopic dermatitis. Allergol Immunopathol.

[32] Diepgen TL, Fartasch M, Hornstein OP (1989). Evaluation and relevance of atopic basic and minor features in patients with atopic dermatitis and in the general population. Acta Derm Venereol Suppl (Stockh).

[33] Dong-Ho N, Myoung-Eun K (2012). Treatment of severe atopic dermatitis with a combination of subcutaneous allergen immunotherapy and cyclosporin. Yonsei Med J.

[34] Jong-Myon B (2014). An overview of systematic reviews of diagnostic tests accuracy. Epidemiol Health.

[35] Scalabrin DM, Bavbek S, Perzanowski MS, Wilson BB, Platts-Mills TA, Wheatley LM (1999). Use of specific IgE in assessing the relevance of fungal and dust mite allergens to atopic dermatitis: a comparison with asthmatic and nonasthmatic control subjects. J Allergy Clin Immunol.

[36] Marsella R, Nicklin C, Lopez J (2005). Atopy patch test reactions in high-IgE beagles to different sources and concentrations of house dust mites. Vet Dermatol.

[37] Imayama S, Hashizume T, Miyahara H, Tanahashi T, Takeishi M, Kubota Y (1992). Combination of patch test and IgE for dust mite antigens differentiates 130 patients with atopic dermatitis into four groups. J Am Acad Dermatol.

[38] Langeveld-Wildschut EG, van Marion AM, Thepen T, Mudde GC, Bruijnzeel PL, Bruijnzeel-Koomen CA (1995). Evaluation of variables influencing the outcome of the atopy patch test. J Allergy Clin Immunol.

[39] Wahlberg JE, Rycroft RJG, Menne T, Frosch PJ, Benezra C (1992). Patch testing. Textbook of Contact dermatitis.

[40] Fisher AA. The role of patch test. In: Fisher AA, editor. Contact dermatitis, 3rd ed. Philadelphia: Lea & Febiger; 1986; p. 25–6.

[41] Turjanmaa K, Darsow U, Niggemann B, Rance F, Vanto T, Werfel T (2006). EAACI/GA2LEN Position paper: present status of the atopy patch test. Allergy.

[42] Nicol NH, Ruzkowski AM, Moore JA (1995). Contact dermatitis and the role of patch testing in its diagnosis and management. Dermatol Nurs.

[43] Sertoli A, Nava C, Angelini G, Meneghini CL, Francalanci S, Gola M (1995). Contact dermatitis of the hands in housewives: an Italian multicentre study. Boll Dermatol Allergol Profess.

